# Rotating axis measurement based on rotational Doppler effect of spliced superposed optical vortex

**DOI:** 10.1515/nanoph-2023-0090

**Published:** 2023-05-11

**Authors:** Xiangyang Zhu, Song Qiu, Tong Liu, You Ding, Ruoyu Tang, Zhengliang Liu, Xiaocen Chen, Yuan Ren

**Affiliations:** Department of Aerospace Science and Technology, Space Engineering University, Beijing 101416, China; Beijing Institute of Tracking and Communication Technology, Beijing 100094, China; Department of Basic Course, Space Engineering University, Beijing 101416, China

**Keywords:** Gouy phase shift, rotating axis azimuth, rotational Doppler effect, spliced superposed optical vortex, topological charge

## Abstract

In most rotational Doppler effect (RDE) measurements, the optical axis and the rotating axis of the object are required to be aligned. However, the condition is very difficult to achieve in practical applications of rotation detection, which seriously affects the received signal. Moreover, it is necessary to focus the beam on the rotating axis of a rotating surface in applications ranging from manufacturing to physical experiments. For example, the manufacture of diffraction optical elements requires aligning the beam to the rotating axis of the spindle. Therefore, how to determine the azimuth of the rotating axis has become an urgent problem to be solved. Based on a new type of superposed vortex beam with multiple topological charges (TCs), we report a new scheme for determining the position of rotating axis by only single RDE measurement, which greatly improves the measurement efficiency. According to the mode decomposition and conservation of angular momentum and energy, we reveal the RDE mechanism of the new structured beam named spliced superposed optical vortex (SSOV) and explain why the SSOV with asymmetrical defect is sensitive to the rotating axis of the object. In addition, in order to prove the effectiveness of the method, a proof-of-concept experiment is conducted to detect the position of object’s rotating axis in eight azimuth ranges, i.e., [*iπ*/4, (*i* + 1)*π*/4](*i* = 0, 1, 2, 3, 4, 5, 6, 7). The idea of breaking the symmetry of the optical vortex (OV) and adding additional parameters in this study may have great potential for applications in optical manipulation and communication. Finally, considering that the orbital angular momentum (OAM) mode purity and quality of the incomplete OV and the SSOV will decrease during the far-field propagation, a new method for pre-correction of SSOV is proposed in this research, which overcomes the effects caused by Gouy phase shift and diffraction to some extent. Combined with inertial navigation, these methods above can also be applied to remote sensing, manufacturing, and physics experiments.

## 1 Introduction

Vortices are common phenomena in nature, ranging from the morphological characteristics of some animals and plants to the spiral distribution structure of galaxies. In recent years, optical vortex (OV) with spiral phase wavefront has attracted widespread attention [1–3]. The phase characteristics of such beams can be expressed by exp(*iℓφ*), where *φ* is azimuth angle and *ℓ* is the orbital angular momentum (OAM) mode index, also known as topological charge (TC). The OV intensity shows a dark hollow envelope structure due to the central phase singularity [4]. At present, OV has been used in many fields, such as rotation detection [5–7], ultra-high-capacity optical communication [8–11], astronomical measurement [12, 13], etc.

The vortex beam with OAM also shows great potential in remote sensing and can be sensitive to the rotational movement along the normal direction [14, 15]. In 1998, Courtial et al. observed the rotational Doppler effect (RDE) which arises from rotational motion and OAM for the first time [16]. Different from the traditional linear Doppler effect (LDE) caused by the relative linear velocity between the object and light source [17], the RDE refers that when the surface of a rotating object is perpendicular to the direction of OV propagation, the rotational motion of the object can also cause frequency shift of the beam. Thus, the RDE provides a powerful way to measure the rotating velocity of a target, which has aroused the great interest of researchers. Subsequently, a large number of studies based on RDE emerge, such as angular acceleration detection [18], compound motion decoupling measurement [19], direction detection of spinning object [20, 21], etc. In 2013, Lavery et al. realized the rotating speed measurement of a spinning object based on the RDE for the first time by using the superposition-mode OV [22]. In 2017, by considering either the motion-induced time-evolving phase or the momentum and energy conservation in light–matter interactions, Liang Fang et al. demonstrated the relationship between RDE and LDE [23]. In 2018, Zhang et al. achieved 120 m long-range RDE detection in the outfield experiment [15], which took an important step for RDE from theory to practical application. However, most speed measurement researches based on RED are limited to the case that the optical axis coincides with the object’s rotating axis. When the OV axis and the rotating axis are not aligned, the frequency spectrum becomes complex, which will harm the experimental results [24]. Meanwhile, in the actual rotating speed detection, it is very difficult to keep the beam axis completely coincident with the rotating axis of an object due to the object’s movement, unknown target position information, and other factors. Therefore, in order to overcome the measurement error caused by misalignment in the experiment and remote sensing, the problem of determining the position of the object’s spinning axis needs to be solved urgently.

At present, most RDE studies use complete OV with circular symmetry as the probe beam, which is insensitive to the changes in circumferential parameters of the object. Even if the vector OV with a non-uniform polarization state is employed, only the rotational speed and direction of the object can be obtained synchronously [25]. The rotational center still can’t be located. The incomplete OV breaks the circular symmetry of the beam but retains the OV characteristics [26], which may acquire some new parameters about the object during the RDE detection and is helpful for solving the problem of determining the rotating center. The previous research [27, 28] about determining the position of the rotating axis by OV and incomplete OV needs to change parameters of the light for multiple measurements, which is very complex. Also, the motion state of the object may have changed during the measurement, which reduces the accuracy of the result.

In this paper, we propose a new type of probe beam named spliced superposed optical vortex (SSOV), which is composed of asymmetric defect OVs with different TCs, to determine the azimuth of rotating axis by only single measurement. First of all, we analyze the OAM spectrum of the SSOV based on the standard Laguerre–Gaussian (LG) beam and explain the relationship between the OAM of the SSOV and the azimuth of the rotating axis. Secondly, combined with the momentum and energy conservation in light–matter interactions, the recognition mechanism of rotating axis azimuth is analyzed in the misaligned condition. And the real-time positioning of the object’s rotating axis has been realized through a single measurement, which greatly shortens the detection time. Then, in order to improve the OAM mode purity of the incomplete OV during propagation, a pre-correction method for incomplete OV and SSOV is proposed. Through this method, the influence of Gouy phase shift [29] can be compensated to a certain extent. Finally, we give an experimental demonstration for the first time, which verifies the correctness and practicability of the theory of determining the object rotating axis by single detection based on the SSOV. This study obtains a new circumferential parameter of rotating object by breaking the circular symmetry of OV and adding multiple TCs. The method in this paper significantly improves the measurement efficiency in remote sensing and has great potential in telemetry, optical manipulation, and astronomical measurement, etc. And the pre-correction method of incomplete OV during propagation may find new applications in the field of RDE remote sensing and communication.

## 2 Theory

### 2.1 Theoretical design and OAM mode analysis of spliced superposed optical vortex

The electric field expression of a complete vortex beam in circular-cylindrical coordinates (*r*, *φ*, *z*) can be written as [30],

(1) $$E_{\text{ov}}\left(r,\varphi ,z\right)=U\left(r,\varphi ,z\right)\exp\left(ikz\right)\exp\left(il\varphi \right)$$

Where *r*, *φ* and *z* are the radius, azimuthal angle, and the distance of propagation of light beam respectively, *k* = *λ*/2*π*, *l*represents TC, *U*(*r*, *φ*, *z*) indicates the amplitude when the propagation distance is *z*. The incomplete OV only retains the sector beam area within the azimuth range Δ*φ* and the light intensity is set to zero in other areas, which breaks the circular symmetry and changes the OAM mode distribution of the beam. Thus, the electric field of incomplete OV can be given by,

(2) $$\begin{array}{c} E_{\text{aov}}\left(r,\varphi ,z\right)=E_{\text{ov}}\left(r,\varphi ,z\right)\left[\varepsilon \left(\varphi -\varphi_{0}\right)-\varepsilon \left(\varphi -\varphi_{0}-\Delta \varphi \right)\right],\\ 0\leq \varphi \leq 2\pi \end{array}$$

where *ɛ*(*φ*) is the step function, *φ*_0_ is the starting azimuth angle of the unobstructed sector of incomplete OV and Δ*φ* represents the azimuth range of the unobstructed sector. Through modulating the phase and TCs of the incomplete OV in different azimuth ranges, the SSOV with different TCs can be obtained. The new type of beam can be seen as a combination of several circularly asymmetric defective OVs with different starting azimuths *φ*_0_, the same azimuthal range Δ*φ*, and different TCs, so we call this beam SSOV. Taking the SSOV with three TCs as an example, the electric field can be expressed as,

(3) $$E_{\text{sov}}\left(r,\varphi ,z\right)=\overset{3}{\underset{n=1}{\sum }}E_{\text{aov}}^{n}\left(r,\varphi ,z\right),\;0\leq \varphi \leq 2\pi$$

where $E_{\text{aov}}^{n}\left(r,\varphi ,z\right)=E_{\text{ov}}^{n}\left(r,\varphi ,z\right)\left[\varepsilon \left(\varphi -\varphi_{n}\right)-\varepsilon \left(\varphi -\varphi_{n}-\Delta \varphi \right)\right]$ , $E_{\text{ov}}^{n}\left(r,\varphi ,z\right)=U\left(r,\varphi ,z\right)\exp\left(\text{i}kz\right)\exp\left(\text{i}l_{n}\varphi \right)$ , *φ*_*n*_ are the starting azimuth angle of incomplete OVs with different TCs in the SSOV, and *φ*_*n*+1_ − *φ*_*n*_ ≥ Δ*φ*. The finally constructed SSOV takes advantage of the asymmetric characteristics of the incomplete vortex beam while keeping different TCs at different azimuths. From Eqs. (2) and (3), it can be seen that the SSOV should have multiple OAM modes because of the introduction of sector defects and multi-TCs.

**Figure 1:**
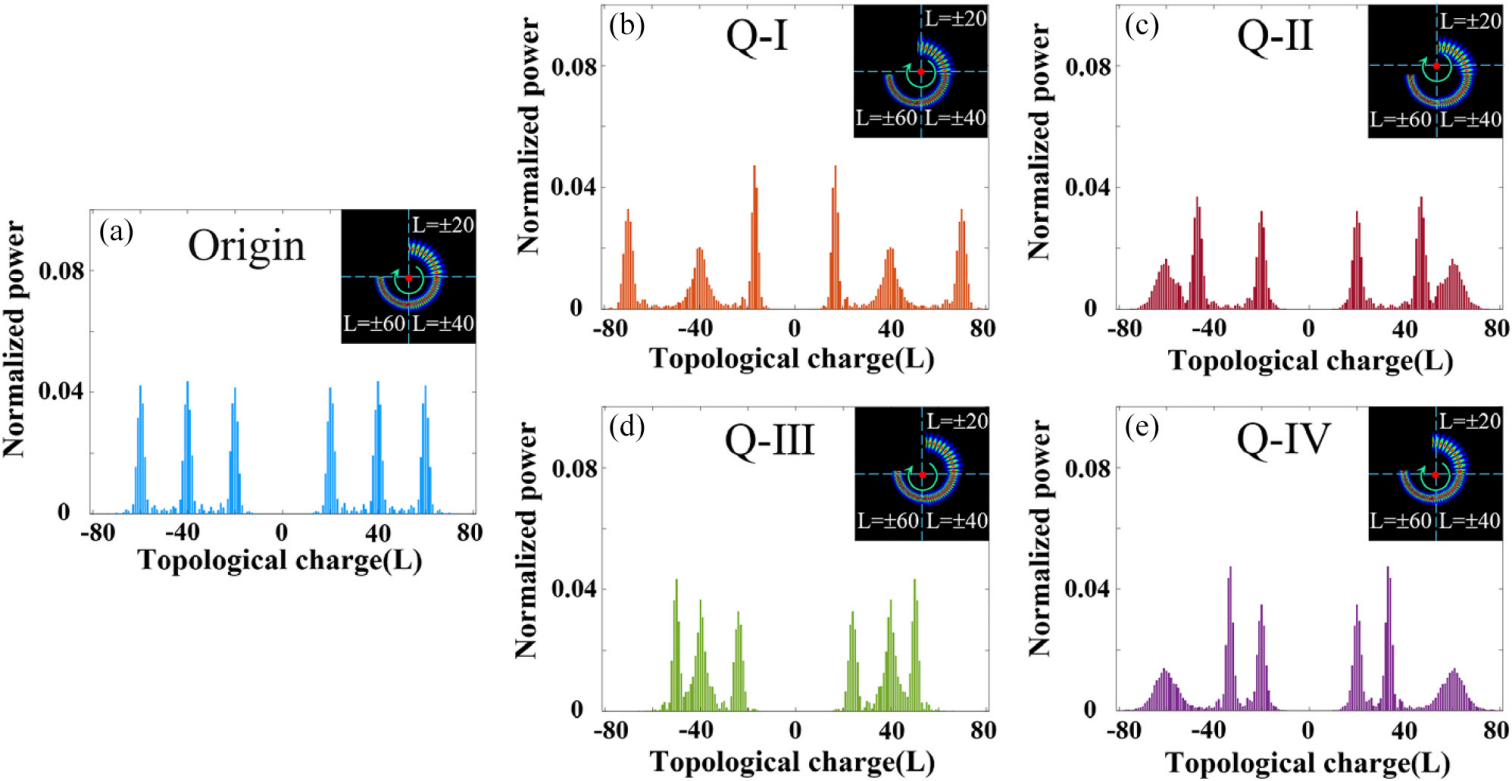
Simulated normalized power spectrums of OAM modes of the SSOV with *l*_1_ = ±20, *l*_2_ = ±60, *l*_3_ = ±40 when the rotating axis lies at different positions. (a) Normalized power spectrum of OAM modes in case the optical axis is aligned with the rotating axis of the object. (b)–(e) Simulated normalized power spectrums of OAM modes in case the object’s rotating axis is located in the four quadrants respectively.

In a quantum system, the wave function of any state can be expanded by a set of orthogonal complete basis vectors. LG beam is an orthogonal and complete basis vector in Hilbert space and any light field can be decomposed into a set of vector states based on the LG mode, including the SSOV. According to the decomposition principle of the OAM mode [31, 32], the SSOV can be represented as the coherent superposition of multiple complete LG beams according to the following functions.

(4) $$E_{\text{sov}}\left(r,\varphi ,z\right)=\overset{+\infty }{\underset{l=-\infty }{\sum }}A_{l,p}E_{\text{LG}}^{l,p}\left(r,\varphi ,z\right)$$

(5) $$A_{l,p}=\overset{2\pi }{\underset{0}{\int }}d\varphi \overset{+\infty }{\underset{0}{\int }}E_{\text{sov}}\left(r,\varphi ,z\right){\left[E_{\text{LG}}^{l,p}\left(r,\varphi ,z\right)\right]}^{*}rdr$$

where $E_{\text{LG}}^{l,p}\left(r,\varphi ,z\right)$ represents a standard LG mode beam whose optical axis coincides with the object’s rotating axis,*A*_*l*,*p*_ is the amplitude related to TC and radial index. Then, based on Eqs. (4) and (5), we perform mode decomposition simulation for the SSOV represented by Eq. (3). And the normalized intensity OAM spectrum only about TC *l* is obtained under the condition that there is lateral misalignment between the optical axis and the rotating axis. First of all, some parameters need to be determined. We set the starting azimuth angles to *φ*_1_ = 0, *φ*_2_ = *π*, *φ*_3_ = 3*π*/2, the sector azimuth range covered by the beam to Δ*φ* = *π*/2, the TCs to *l*_1_ = ±20, *l*_2_ = ±60, *l*_3_ = ±40. The azimuth angle (*θ*) of the optical axis is set to *iπ*/4(*i* = 1, 3, 5, 7) to simulate the different positions of propagation axis of the SSOV, which is equivalent to fixing the optical axis and placing the object rotation axis in different directions. The simulated OAM spectrums are shown in Figure 1.

Figure 1(a) shows that when the rotating axis of the object is aligned with the optical axis, the OAM spectrum has three parts of the broadening range, the central peaks correspond to different TCs, i.e., *l*_1_ = ±20, *l*_2_ = ±60, *l*_3_ = ±40 respectively, and the OAM mode interval is 1. The OAM spectrum still shows a three-part mode region, but the central peak and expanded range vary with the azimuth angle (*θ*) of the optical axis when there is lateral misalignment between the beam axis and the object’s rotating axis, as shown in Figure 1(b)–(e). The variation of OAM spectrum with the azimuth angle (*θ*) is shown in Table 1, which indicates that the mode spectrum of the SSOV is sensitive to the azimuth of the object’s rotating axis. It is obvious that the RDE frequency shift is proportional to the OAM mode based on the theoretical rotational Doppler frequency shift *l*Ω/2*π*. Therefore, we can employ the SSOV to determine the position of the rotating axis through a single RDE measurement. When the rotating axis is in the first quadrant, the absolute value of three-part OAM spectrum of the SSOV are less than 20, i.e., $|l_{1}^{\prime }|<20$ more than 60, i.e., $|l_{2}^{\prime }|>60$ , and expanding around 40, i.e., $|l_{3}^{\prime }|∼40$ , respectively. And so on for other quadrants.

**Table 1:** The variation of OAM spectrum. $l_{1}^{\prime }$ , $l_{2}^{\prime }$ , $l_{3}^{\prime }$ represents the central peaks of the three-part OAM spectrum of the SSOV obtained by the mode decomposition method. The left column denotes the azimuth of the rotating axis of the object.

Quadrant	OAM		
	$\begin{array}{c} \left|{l^{\prime }}_{1}\right|\\ \left(l_{1}=\pm 20\right) \end{array}$	$\begin{array}{c} \left|{l^{\prime }}_{2}\right|\\ l_{2}=\pm 60 \end{array}$	$\begin{array}{c} \left|{l^{\prime }}_{3}\right|\\ l_{3}=\pm 40 \end{array}$
Origin	=|*l*_1_|	=|*l*_2_|	=|*l*_3_|
Q-I(*θ* = *π*/4)	$<|l_{1}|$	$>|l_{2}|$	∼|*l*_3_|
Q-II(*θ* = 3*π*/4)	∼|*l*_1_|	∼|*l*_2_|	$>|l_{3}|$
Q-III(*θ* = 5*π*/4)	$>|l_{1}|$	$<|l_{2}|$	∼|*l*_3_|
Q-IV(*θ* = 7*π*/4)	∼|*l*_1_|	∼|*l*_2_|	$<|l_{3}|$

### 2.2 Principle of the rotating axis orientation measurement

Then from the conservation of the angular momentum and energy, we quantitatively analyze the effect of the position of rotating axis on the rotational Doppler frequency shift caused by the SSOV. In the light–matter interactions, the rotating particle at rotational velocity Ω_1_ does work against this torque of *lℏ* per photon delivering an energy to the reflected light of Δ*E* = *lℏ*Ω_1_ cos(*γ*) = *lℏ*Ω_0_ per photon, leading to a rotational Doppler shift, which can be expressed as [23],

(6) $$\Delta f=\Delta E/\hbar /2\pi =l\Omega_{0}/2\pi$$

where Ω_0_ represents the angular velocity delivered to the reflected photon and is the tangential velocity component of the rotating particle relative to the vortex beam. However, things will be different if the SSOV illuminates the rotating object non-coaxially. When there is lateral misalignment between the rotating axis and the optical axis, the energy Δ*E* delivered by every spinning particle to the reflected photons in the light field is different, and this will produce multiple peaks in the RDE frequency spectrum.

**Figure 2:**
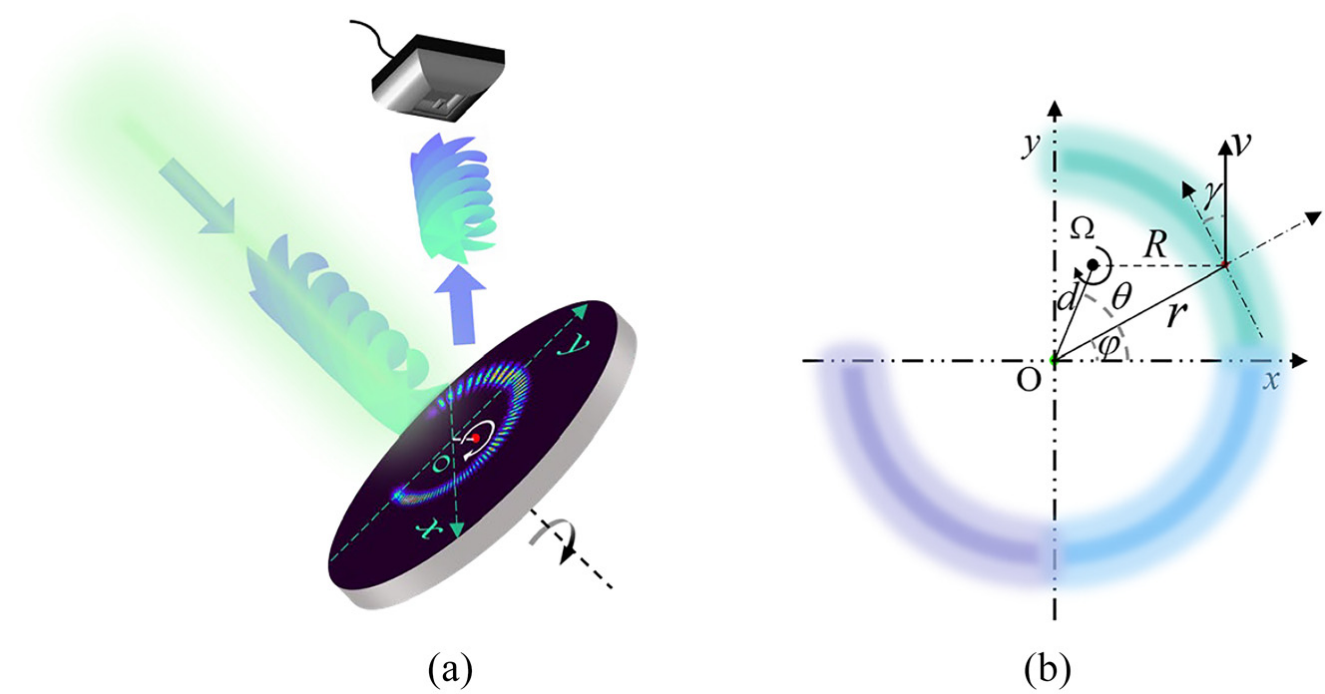
Schematic diagram of the RDE under misaligned illumination condition. (a) The SSOV irradiates the rotating object under the misaligned condition and the Cartesian coordinate system is established with the spot center as the origin. The red point represents the object’s rotating center. (b) The simplified front view along propagation direction of the beam.

In order to further analyze the rotational Doppler frequency shift of echo signal under misaligned condition, we establish the Cartesian coordinate system with the center of the SSOV as the origin, as shown in Figure 2(b). The horizontal and vertical directions of the beam cross-section are set as the *x*-axis and *y*-axis. The angle of each small rotating particle in the light field is expressed by *φ*, the position of the object’s rotating axis is defined by *θ*, and *γ* is the angle between the linear velocity of the rotating particle and the tangent direction of the beam. *d* is the distance between the object’s rotating center and the origin, *r* is the radius of the SSOV, and *v* is the linear velocity of the particle. Based on the geometric relations in Figure 2(b), we can get the equation of *φ*, *θ*, *γ*.

(7) $$\left\{\begin{array}{c} d^{2}=R^{2}+r^{2}-2Rr\cos\left(\gamma \right)\;\\ R^{2}=r^{2}+d^{2}-2rd\cos\left(\varphi -\theta \right)\; \end{array}\right.$$

From Eq. (7), we can get the angle (*γ*) between the linear velocity of the object particle and the tangential direction of the OV. Then we decompose the linear velocity *v* along the tangent direction and the radial direction of the OV cross-section. The angular velocity Ω_0_ of the rotating particles delivered to the reflected light can be given by,

(8) $$\Omega_{0}=\frac{v\cos\left(\gamma \right)}{r}=\frac{\Omega \left[r-d\cos\left(\varphi -\theta \right)\right]}{r}$$

The final RDE frequency shift generated by scattered light is,

(9) $$\Delta f=\frac{l\Omega }{2\pi }\cdot \frac{r-d\cos\left(\varphi -\theta \right)}{r}$$

Based on Eq. (9), when the SSOV expressed by Eq. (3) is used to illuminate a rotating target, the total RDE frequency shift is,

(10) $$\Delta f=\left\{\begin{array}{c} \frac{l_{1}\Omega }{2\pi }\cdot \frac{r-d\cos\left(\varphi -\theta \right)}{r},\;\varphi_{1}\leq \varphi \leq \varphi_{1}+\Delta \varphi \;\\ \frac{l_{2}\Omega }{2\pi }\cdot \frac{r-d\cos\left(\varphi -\theta \right)}{r},\;\varphi_{2}\leq \varphi \leq \varphi_{2}+\Delta \varphi \;\\ \frac{l_{3}\Omega }{2\pi }\cdot \frac{r-d\cos\left(\varphi -\theta \right)}{r},\;\varphi_{3}\leq \varphi \leq \varphi_{3}+\Delta \varphi \;\\ 0,\;other\; \end{array}\right.$$

According to Eq. (9), it can be clearly seen that when a complete OV is used to illuminate a rotating object, the RDE frequency shift range is $\left[\frac{l\Omega }{2\pi }\cdot \frac{r-d}{r},\frac{l\Omega }{2\pi }\cdot \frac{r+d}{r}\right]$ . Thus, the expanded RDE frequency spectrum is independent of the azimuth of the rotating axis *θ* when *l*, Ω, *d* are fixed. Therefore, it is impossible to determine the rotating axis of the object by employing the complete OV. When the SSOV is used, the azimuth angle range [*φ*_*i*_, *φ*_*i*_ + Δ*φ*]of the SSOV is constant. At this time, the broadened frequency spectrum in Eq. (10) is only related to *θ* in the cosine function, so the SSOV can be sensitive to the azimuth of the rotating axis based on RDE.

In the experiments and simulations, we use the superposition-mode SSOV as the probe beam which can greatly reduce the requirements of the experimental setup and light path adjustment. In addition, under inclined irradiation conditions, the linear Doppler shift can be counteracted and the rotational Doppler shift is doubled by using the superposition state of the vortex beam with positive and negative TCs. Therefore, the superposition of the vortex beam is a better choice for rotational measurement.

**Figure 3:**
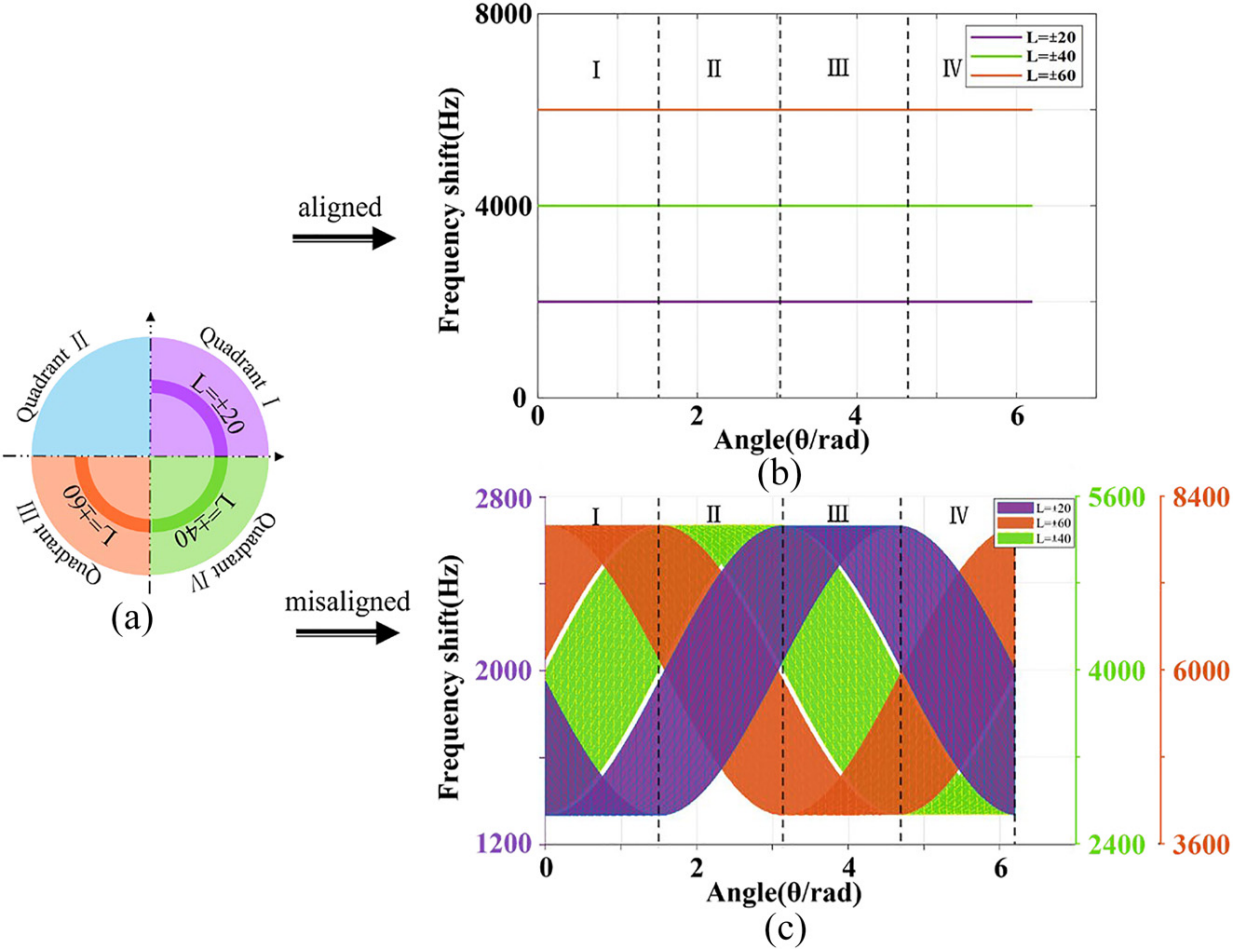
Simulated results of the RDE frequency shift corresponding to SSOV with *l*_1_ = ±20, *l*_2_ = ±60, *l*_3_ = ±40. The horizontal axis and the vertical axis represents the azimuth of the rotating axis and RDE frequency shift respectively. (a) The SSOV with different TCs. (b) The total RDE frequency shift generated by the SSOV under the aligned condition. (c) The total RDE frequency shift generated by the SSOV under the misaligned condition. Stripes with Orange, green, purple represent the frequency spectrums corresponding to the incomplete OVs with *l*_1_ = ±20, *l*_2_ = ±60, *l*_3_ = ±40, in the SSOV respectively.

To further explain the principle of the azimuth discrimination of the rotating axis, we simulated the distribution of RDE frequency shift. Firstly, the TCs of the new SSOV are set to *l*_1_ = ±20, *l*_2_ = ±60, *l*_3_ = ±40 and the corresponding starting azimuthal angles of incomplete OV are set to *φ*_1_ = 0, *φ*_2_ = *π*, *φ*_3_ = 3*π*/2. The azimuth interval is set to Δ*φ* = *π*/2, the rotational speed is set to Ω = 50 Hz, the radius is set to *r* = 3 mm, and the misalignment distance is set to *d* = 1 mm. Based on Eq. (10), we numerically simulated the RDE frequency shift of all scattered light at different azimuths of the SSOV field under the aligned and unaligned conditions respectively, as shown in Figure 3.

When the SSOV is used for RDE detection, the rotational Doppler frequency shift obtained is three constant values, namely 2000 Hz, 4000 Hz, and 6000 Hz on the condition of coaxial incidence, as shown in Figure 3(b). Under noncoaxial illumination condition, the total RDE frequency shift generated by the SSOV fluctuates with the azimuth of rotating axis *θ* and shows three different frequency spectral bands with the frequency shift of 2000 Hz, 4000 Hz and 6000 Hz respectively as the basis, as shown in Figure 3(c). This is because the RDE frequency shift caused by the SSOV under the unaligned condition depends on the azimuth (*φ*) of unobstructed beam, TC (*l*) and the azimuth (*θ*) of the object’s rotating axis. Then when the first two of the variables are fixed, the RDE frequency spectrum is only related to *θ* so that we can determine the quadrant in which the rotating axis is located.

Considering that the rotating axis of the object is located in the quadrant I, namely 0 ≤ *θ* ≤ *π*/2. The three-part rotational Doppler frequency shifts obtained by one measurement are less than 2000 Hz, that is, $f_{1}^{\prime }<2000\;Hz$ , more than 6000 Hz, that is, $f_{2}^{\prime }>6000\;Hz$ , and spreading around 4000 Hz, that is, $f_{3}^{\prime }∼4000\;Hz$ , respectively. When the rotating axis is located in other quadrants, the total RDE frequency shift related to the SSOV is also different, as shown in Table 2. The theoretical analysis and this simulation results prove the feasibility of the rotating axis position resolution method.

**Table 2:** Azimuth resolution method of rotating axis in 4 quadrants. $f_{1}^{\prime }$ , $f_{2}^{\prime }$ , $f_{3}^{\prime }$ represents the middle values of the three-part rotational Doppler shifts obtained in one measurement. The left column denotes the azimuth of the rotating axis of the object, i.e., *θ* ∈ (*iπ*/2, (*i* + 1)*π*/2), *i* = 0, 1, 2, 3.

Quadrant	Δ*f*		
	$\begin{array}{c} {f^{\prime }}_{1}\\ \left(f_{1}=2000\;\text{H}\text{z}\right) \end{array}$	$\begin{array}{c} {f^{\prime }}_{2}\\ \left(f_{2}=6000\;\text{H}\text{z}\right) \end{array}$	$\begin{array}{c} {f^{\prime }}_{3}\\ \left(f_{3}=4000\;\text{H}z\right) \end{array}$
Origin	=*f*_1_	=*f*_2_	=*f*_3_
Q-I	$<f_{1}$	$>f_{2}$	∼*f*_3_
Q-II	∼*f*_1_	∼*f*_2_	$>f_{3}$
Q-III	$>f_{1}$	$<f_{2}$	∼*f*_3_
Q-IV	∼*f*_1_	∼*f*_2_	$<f_{3}$

**Figure 4:**
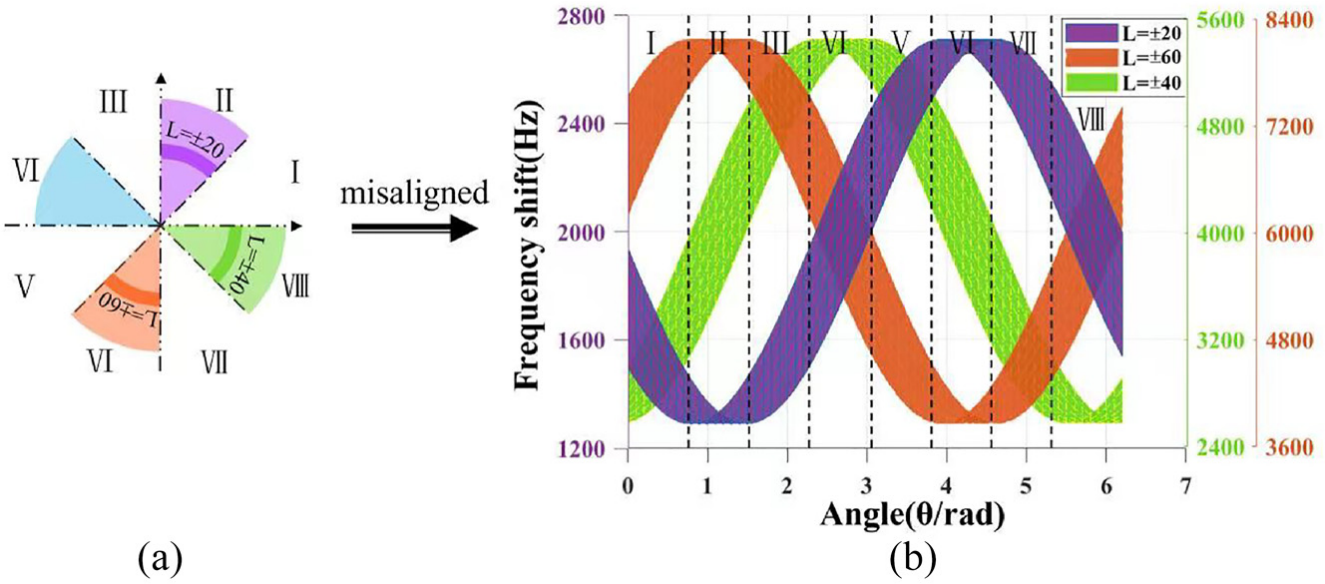
Simulated results of the RDE frequency shifts generated by the SSOV with Δ*φ* = *π*/4. (a) The SSOV with the coverage azimuth range of *π*/4. (b) Theoretical RDE frequency shift when the SSOV is employed as probe beam under the misaligned condition. The azimuth angle (*θ*) of rotating axis is divided into 8 parts, i.e., *θ* ∈ (*iπ*/4, (*i* + 1)*π*/4), *i* = 0, 1, 2, 3, 4, 5, 6, 7.

In addition, we can also employ the SSOV with smaller coverage range and more different TCs as the probe light, such as the SSOV with azimuth coverage range of *π*/4, as shown in Figure 4(a). We set the initial azimuth of to *φ*_1_ = *π*/4, *φ*_2_ = 5*π*/4, *φ*_3_ = 7*π*/4. The rest parameters are the same as above. And then we simulated the RDE frequency shift when this SSOV with Δ*φ* = *π*/4 is used as probe beam. The results are shown in Figure 4(b).

The simulation results show that the three-part frequency spectrums generated by the SSOV are different when the rotating axis is located in different positions, i.e., *θ* ∈ (*iπ*/4, (*i* + 1)*π*/4), *i* = 0, 1, 2, 3, 4, 5, 6, 7. According to this scheme, we can achieve the resolution of the rotating axis in 8 azimuth ranges under the unaligned condition. The specific distinguishing method is shown in Table 3. Taking the rotating axis in the first azimuth range [0, *π*/4] as an example, the three-part rotational Doppler frequency shifts obtained by one measurement are less than 2000 Hz, that is, $f_{1}^{\prime }<2000\;Hz$ , more than 6000 Hz, that is, $f_{2}^{\prime }>6000\;Hz$ , and less than 4000 Hz, that is, $f_{3}^{\prime }<4000\;Hz$ , respectively. And so on for other quadrants.

**Table 3:** Azimuth recognition method of rotating axis in 8 azimuth ranges. $f_{1}^{\prime }$ , $f_{2}^{\prime }$ , $f_{3}^{\prime }$ represents the middle values of the three-part rotational Doppler shifts obtained in one measurement. The left column denotes the azimuth of the rotating axis of the object, i.e., *θ* ∈ (*iπ*/4, (*i* + 1)*π*/4), *i* = 0, 1, 2, 3, 4, 5, 6, 7.

Azimuth	Δ*f*		
range (*θ*)			
	$\begin{array}{c} {f^{\prime }}_{1}\\ \left(f_{1}=2000\;\text{H}\text{z}\right) \end{array}$	$\begin{array}{c} {f^{\prime }}_{2}\\ \left(f_{2}=6000\;\text{H}\text{z}\right) \end{array}$	$\begin{array}{c} {f^{\prime }}_{3}\\ \left(f_{3}=4000\;\text{H}\text{z}\right) \end{array}$
Origin	=*f*_1_	=*f*_2_	=*f*_3_
I	$<f_{1}$	$>f_{2}$	$<f_{3}$
II	$<f_{1}$	$>f_{2}$	∼*f*_3_
III	$<f_{1}$	$>f_{2}$	$>f_{3}$
IV	∼*f*_1_	∼*f*_2_	$>f_{3}$
V	$>f_{1}$	$<f_{2}$	$>f_{3}$
VI	$>f_{1}$	$<f_{2}$	∼*f*_3_
VII	$>f_{1}$	$<f_{2}$	$<f_{3}$
VIII	∼*f*_1_	∼*f*_2_	$<f_{3}$

## 3 Experiment and results

### 3.1 Experimental methods

**Figure 5:**
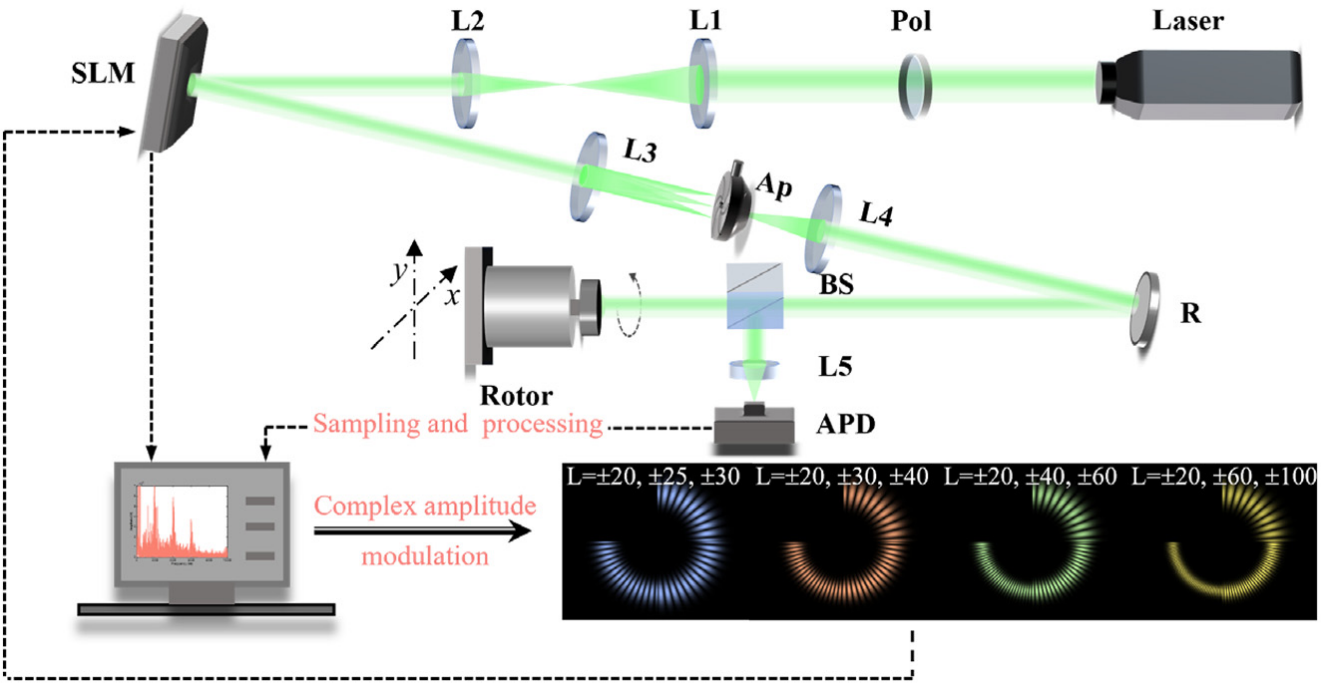
Experimental setup. Pol, polarizer. L1, L2, L3 and L4, lens with a focal length of 100 mm. L5, len with a focal length of 50 mm. SLM, spatial light modulator. Ap, Aperture. R, reflector. BS, beam splitter. APD, avalanche photodetector. The computer is used to process the data and adjusts the holograms. The SSOVs with different TC intervals generated by the complex amplitude modulation method.

In order to verify the correctness of theoretical analysis on azimuth recognition of the rotating axis, a proof-of-concept experiment is designed, as shown in Figure 5. The intensity profiles with different colors in Figure 5 represent the SSOVs with TC intervals of 5, 10, 20, and 40 respectively.

The 532 nm laser beam generated by the solid-state laser passes through the polarizer (Pol) and is converted into linearly polarized state. Then after being expanded and collimated by the 4f system composed of L1 and L2, the probe beam illuminates the SLM loaded with the SSOV hologram. The SSOV is generated by diffraction. We filter out the first-order diffracted light with the best intensity and mode purity by L3, Aperture (Ap), and L4. After passing the reflector(R) and beam splitter (BS), the SSOV irradiates the rotating object. Finally, the scattered light is collected and sampled by the avalanche photodetector (APD). The computer in Figure 5 is responsible for processing the optical signals collected by the APD and modulating the holograms. In order to improve signal-to-noise ratio, we stick the metal reflective material on the surface of the rotor to ensure that the RDE signal is strong enough. The position of the rotor can be precisely controlled through the adjustable frame and the rotor speed is regulated by the controller.

In terms of hologram design, we use complex amplitude modulation method to flexibly modulate the phase and amplitude, and obtain different SSOV holograms. Then the required SSOVs can be generated by diffraction. At the same time, in order to avoid the hybrid of rotational Doppler frequency spectrum caused by beam overlap, the initial azimuth intervals (*φ*_*i*+1_ − *φ*_*i*_) of incomplete OVs in SSOV should be more than the unobstructed azimuth range (Δ*φ*).

### 3.2 Results and discussion

**Figure 6:**
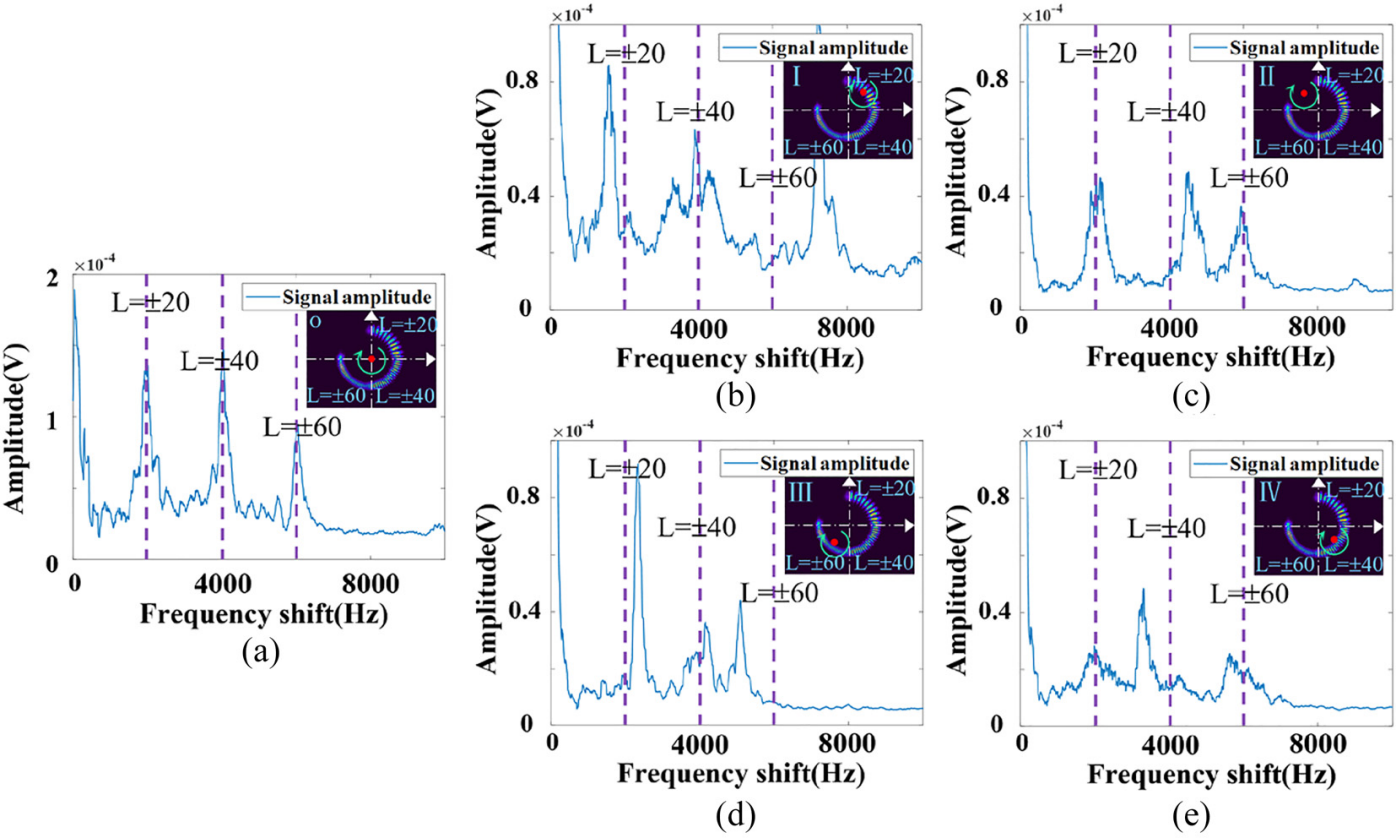
Experiment results under the illumination of SSOV with Δ*φ* = *π*/2. The red dot represents the rotating center of the object, the purple dotted line expresses center frequency shifts of 2000 Hz, 4000 Hz, 6000 Hz corresponding to the TCs, *L* = ±20, *L* = ±40, *L* = ±60. o indicates the origin. I, II, III, IV respectively represent different quadrants of the coordinate system. (a) Experimental RDE frequency spectrum under alignment condition. (b)–(e) Experimental RDE frequency spectrums when rotating axis of the object is in the four quadrants.

We first divide the whole SSOV field into four quadrants in the experiment. Then we set the TCs of the SSOV to *l*_1_ = ±20, *l*_2_ = ±60, *l*_3_ = ±40 and corresponding coverage azimuth ranges to [0, *π*/2], [*π*, 3*π*/2], [3*π*/2, 2*π*] respectively. The beam radius and the lateral misalignment distance are set to 3 mm and 1 mm. The rotating speed of the object is set to 50 rps. We use the SSOV with Δ*φ* = *π*/2 to illuminate the object’s surface on the conditions of alignment and misalignment respectively. Then we employ APD to capture the time-domain echo signal within 1 s. After Fourier transform, the rotational Doppler frequency spectrum is obtained as shown in Figure 6.

Figure 6(a) shows the RDE frequency shift distribution when the rotating axis is aligned with the optical axis. When the SSOV illuminates the surface of the rotating body coaxially, the rotational Doppler frequency spectrum is slightly expanded around the central frequency shift 2000 Hz, 4000 Hz, 6000 Hz. This is because the asymmetric defect of the incomplete OVs in the SSOV field leads to the broadened OAM spectrum, but the central OAM is still consistent with the set value, as shown in Figure 1(a). Therefore, the OAM-related RDE frequency spectrum shows the same variation. Figure 6(b)–(e) represents the RDE frequency shifts when the rotating axis lies in quadrant I, II, III, IV. It can be seen evidently that the frequency spectrums vary with the position of the rotating axis. When the rotating axis of the object is located in quadrant I, the three-part RDE frequency shifts corresponding to the superposed OVs with different TCs in the SSOV obtained by one measurement are less than 2000 Hz, i.e., $f_{l_{1}}^{\prime }<2000\;Hz$ , more than 6000 Hz, i.e., $f_{l_{2}}^{\prime }>6000\;Hz$ , and expanding around 4000 Hz, i.e., $f_{l_{3}}^{\prime }∼4000\;Hz$ , respectively. And so on for other quadrants. The same goes for the other quadrants. The experimental results are in good agreement with the theoretical analysis, as shown in Table 2.

**Figure 7:**
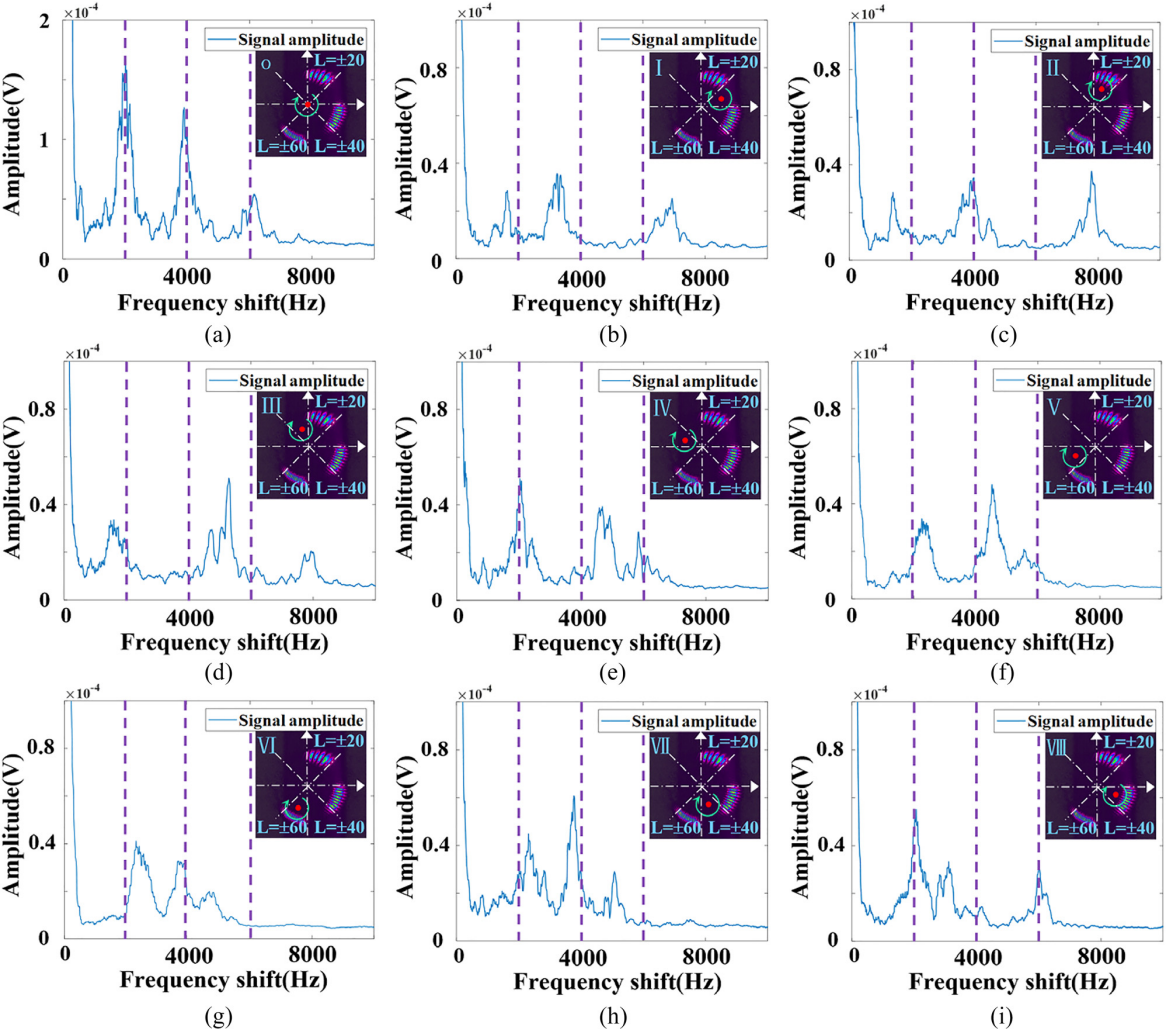
Experiment results under the illumination of SSOV with Δ*φ* = *π*/4. (a) RDE frequency spectrum under alignment condition. (b)–(i) RDE frequency spectrum when rotating axis of the object is in each part of the 8 azimuth ranges.

Under the same experimental conditions, we perform RDE experiments using SSOV with a smaller azimuth coverage range of Δ*φ* = *π*/4 as probe beam. The experimental results are shown in Figure 7, which show that the mechanism of the position measurement of the rotating axis based on RDE frequency spectrums is in good agreement with the theoretical analysis. The distribution of RDE frequency spectrum will change with the position of the rotation axis accordingly and is unique. Taking the first azimuth range [0, *π*/4] as an example, in the total RDE frequency spectrum, the frequency shift corresponding to the incomplete OV with *l*_1_ = ±20 is less than 2000 Hz, i.e., $f_{l_{1}}^{\prime }<2000\;Hz$ , the frequency shift corresponding to the incomplete OV with *l*_2_ = ±60 is more than 6000 Hz, i.e., $f_{l_{2}}^{\prime }>6000\;Hz$ , and the frequency shift corresponding to the incomplete OV with *l*_3_ = ±40 is less than 4000 Hz i.e., $f_{l_{3}}^{\prime }<4000\;Hz$ . Similarly, the specific method for determining the azimuth of the rotating axis is shown in Table 3. Therefore, we have successfully achieved the resolution of the object’s rotating axis in eight azimuth ranges, *θ* ∈ (*iπ*/4, (*i* + 1)*π*/4), *i* = 0, 1, 2, 3, 4, 5, 6, 7.

However, it is worth noting that when the coverage range (Δ*φ*) of the beam in the SSOV field is smaller, although the azimuth of the rotating axis can be divided more precisely, the expansion of OAM mode spectrum caused by the asymmetric defect is more serious. This will lead to severe interference and make the RDE frequency spectrum more chaotic and complex. By comparing Figures 6 and 7, it can be found that resolution of the object’s rotating axis in the four quadrants is clearer than that in the 8-azimuth ranges. Therefore, accurate positioning of the rotating axis at each azimuth angle depends on more precise light field modulation and optimized signal processing methods.

Further, combined with Eq. (10), the precise position of rotating axis of an object can be measured. We choose the middle values (Δ*f*_*i*_) of the three-part rotational Doppler shift band obtained from one measurement respectively. When the TC (*l*_*i*_), rotational speed (Ω), radius of the vortex beam (*r*), and azimuth angle (*φ*) are determined, not only the azimuth angle of rotating axis (*θ*) but also the misalignment distance (*d*) can be calculated precisely in the actual detection. The rotational speed (Ω) can be obtained by extracting the interval among the peaks of the rotational Doppler shift [33], and the azimuth angle (*φ*) is equal to *φ*_*i*_ + Δ*φ*/2 related to the central rotational Doppler shift (Δ*f*_*i*_). In this paper, we mainly focus on the azimuth of the rotating axis, so we do not discuss the offset distance in detail, which is what we need to seriously consider next.

Nevertheless, some errors still exist in the measurement of the azimuth of the rotating axis due to data processing, device and other reasons, such as data filtering process. In the experiment, the frequency resolution of the sampled data is 1 Hz, and then a smoothing filter with a window width of 160 is used to optimize the signal. So, the total frequency shift error introduced by data processing is 160 Hz. Combined with Eq. (11) in the paper, the maximum measurement error of the rotating axis azimuth is 4.46° under the conditions that beam radius is 3 mm (*r* = 3 mm), lateral offset distance equals 1 mm (*d* = 1 mm), and beam azimuth is*π*/4 (*φ* = *π*/4). In addition, the vibration of the rotor, the thermal noise of the detector, and other disturbances will also have an impact on the accuracy of the rotating axis orientation measurement. The above analysis shows that the measurement error of the azimuth angle of the rotating axis is not fixed and attributed to various factors such as topological charge, beam radius, misaligned distance, rotating axis azimuth and so on.

**Figure 8:**
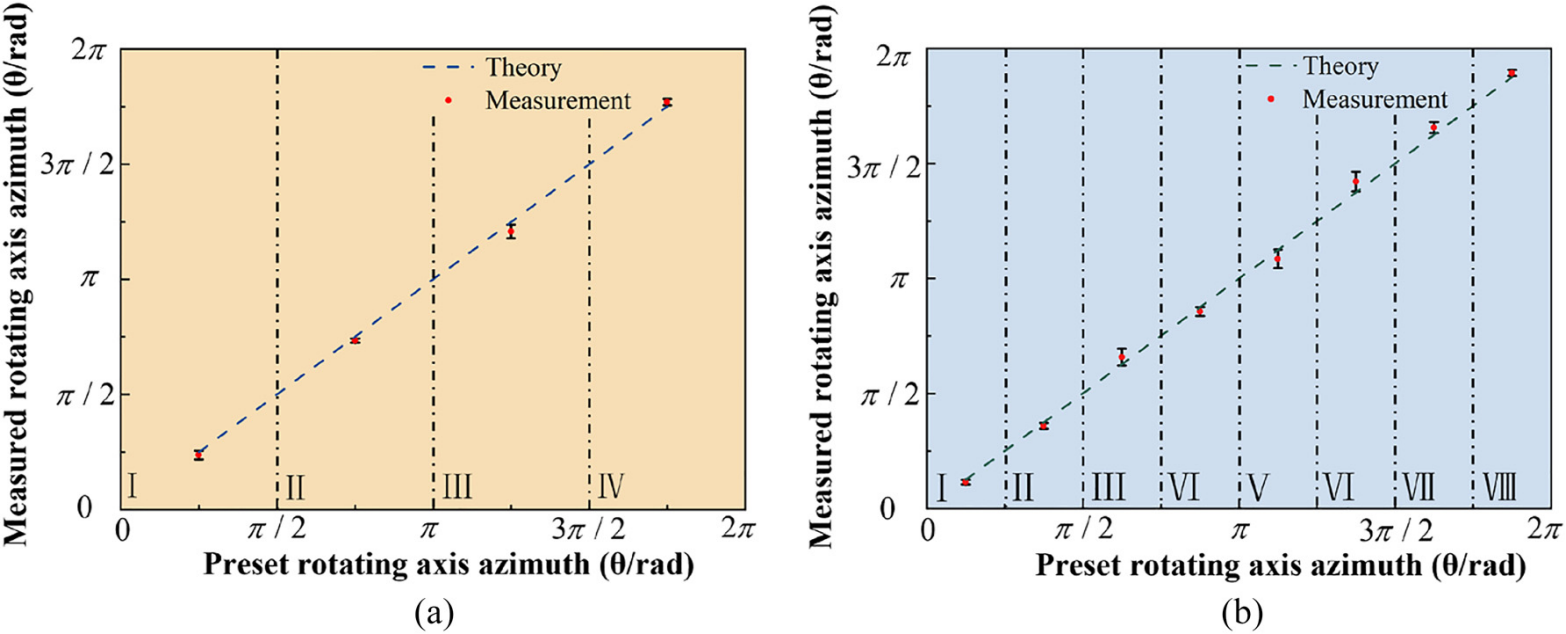
Measurement results and error analysis. (a) Measurement results and errors of the azimuth of rotating axis in four quadrants. (b) Measurement results and errors of the azimuth of the rotating axis in eight azimuthal ranges.

Combining with RDE, we further analyzed the measurement error of the azimuth of the rotating axis for the experimental data based on Eq. (10). And the results are shown in Figure 8.

Figure 8 shows that the measured value of the rotating axis position is in good agreement with the set value within the acceptable error range, which proves the effectiveness of the rotational axis measurement method based on the SSOV. In practical engineering, if high accuracy is required, we can precisely measure the position of the rotating axis according to Eq. (10) within a certain error range, otherwise, we can quickly and conveniently determine the azimuth range through the method of RDE frequency shift comparison.

**Figure 9:**
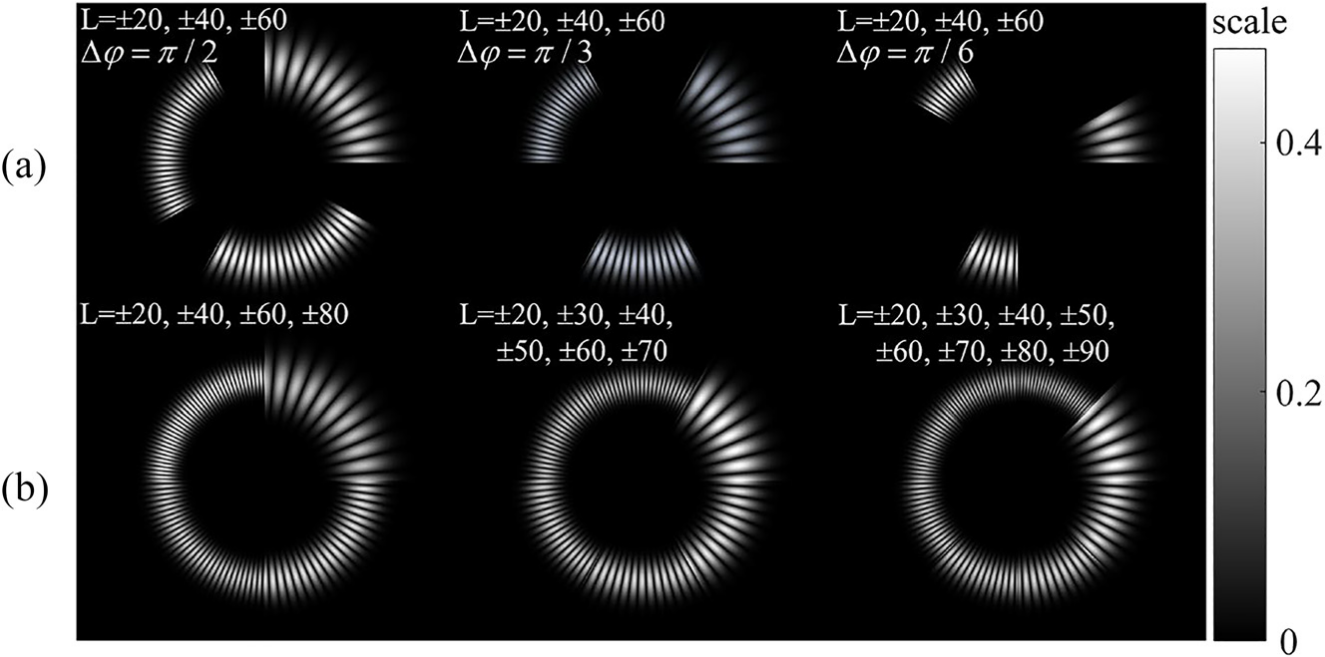
SSOVs with different types. (a) SSOVs with a certain azimuth gap. (b) SSOVs with multiple TCs.

Although the above scheme can be used to determine the position of the object’s rotating axis through a single RDE detection, which greatly improves the detection efficiency, the misalignment distance set in the experiment is small. Combined with formula (9), the spreading effect of RDE frequency spectrum is more severe when the misalignment distance (*d*) is further increased. And at the same time, the three central RDE frequency shift intervals Δ*l*Ω/2*π* will be further reduced when the rotating speed (Ω) of the object decreases. These uncontrollable factors will make the distributions of three-part RDE frequency shift aliased and unclear to distinguish from each other in the actual measurement. These problems need to be further solved. In addition, it is possible to use the SSOVs with more different TCs and smaller azimuth range Δ*φ*, as shown in Figure 9(b), which achieves more precise division of the position of rotating axis of an object. In remote sensing, the long-distance propagation of SSOV will cause the incomplete superposed OVs with different TCs in SSOV to interfere with each other at the junction. Therefore, during the generation of SSOVs, a certain azimuth gap is required among beams with different TCs to reduce interference, as shown in Figure 9(a). In recent years, Zhai et al. proposed the radial Doppler effect [34], which can measure the radial velocity of particles but cannot determine the direction of particle movement. Adding different “TCs” at different azimuths in this paper may help solve this problem.

**Figure 10:**
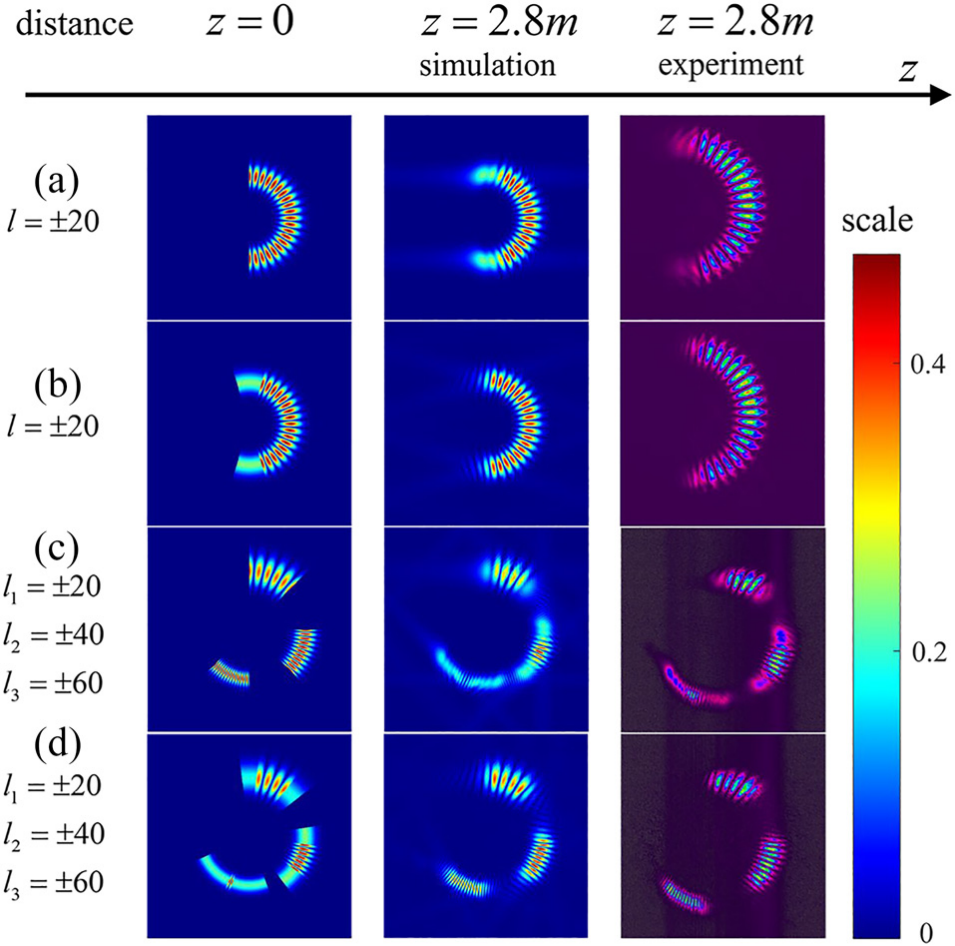
Calculated and experimentally-observed spatial evolution of modified incomplete OV and modified SSOV. (a) and (c) Simulation and experimental observation results of the uncorrected OV and SSOV at propagation distance of 2.8 m. (b) and (d) Simulation and experimental observation results of the modified OV and SSOV at propagation distance of 2.8 m.

Due to the influence of Gouy phase shift and diffraction, the rotating directions of incomplete OVs with +TC and −TC are opposite during propagation [35], which makes the self-interference effect gradually weaken and reduces the OAM mode purity of the beam. The edge distortion of incomplete OVs is also serious, which greatly affects the RDE detection effect and the measurement accuracy of position of the rotating axis. Therefore, in order to overcome these shortcomings, this paper proposes a new correction method for the distortion of incomplete superposed OVs and SSOVs. The Gouy phase is given by [29],

(11) $$\phi =\left(2p+|l|+1\right)\arctan\frac{z}{z_{\text{R}}}$$

where, $z_{\text{R}}=kw_{0}^{2}/2$ is Rayleigh distance, *p* is the radial index, *l* represents TC, *k* is wave number, expressed as 2*π*/*λ*, *λ* is beam wavelength, and *z* is propagation distance. During the generation of incomplete superposed OV, we add the phase-compensated angle to the hologram in advance based on Eq. (11). Then an incomplete OV that is not completely superimposed can be obtained. This particular incomplete OV with +TC and −TC rotates during propagation until it overlaps completely when hitting the target, as shown in Figure 10(b) and (d). Because the SSOVs are composed of incomplete superposed OVs, this method can also be applied to the pre-correction of SSOVs. We simulated the modified incomplete-OV and the modified SSOV at the propagation distance of 2.8 m, and observed their intensity profiles by CCD in the experiment. The results, shown in Figure 10, demonstrate the validity of this pre-correction method. Compared to the uncorrected superposed vortex beams at the same propagation distance, the quality of the pre-corrected beams is improved.

In addition, for long-distance propagation of the SSOV, the rotating phase angle tends to the limit value *π*/2 because of the influence of Gouy phase shift. Thus, the deflection phase angle *π*/2 can be directly added to the hologram of the SSOV beforehand when the propagation distance is too far. By this method, the quality of the incomplete OV during propagation can be improved, which has great application potential in remote sensing and information transmission.

## 4 Conclusions

The paper proposes a new measurement scheme for the position of the object’s rotating axis. In this scheme, by adding multiple TCs and breaking the circular symmetry of OVs, the SSOV is constructed. We establish the coordinate system with the center of the SSOV as the origin, and divide the light field into eight azimuth angle ranges [*iπ*/4, (*i* + 1)*π*/4](*i* = 0, 1, 2, 3, 4, 5, 6, 7). Then employing SSOV as a probe beam, we have successfully measured the position of the rotating axis in eight azimuth ranges through a single light–matter interaction. Theoretically, according to Eq. (10), not only the azimuth angle of rotating axis (*θ*) but also the misalignment distance (*d*) can be calculated precisely in the actual detection. However, in the actual telemetry, there are measurement errors due to data processing, device, and other reasons. If high accuracy is required, we can precisely measure the position of the rotating axis according to Eq. (10) within a certain error range, otherwise, we can quickly and conveniently determine the azimuth range through the method of RDE frequency shift comparison. Furthermore, it is expected to achieve more precise division by using the SSOVs with smaller azimuth intervals (Δ*φ*) and better data processing methods.

At the same time, considering the influence of Gouy phase shift and diffraction for the SSOV in free space, this study proposes a method for improving the OAM mode purity of the SSOV. Because the vortex beams with +TC and −TC have different rotating directions in propagation. Therefore, we add phase-compensated angle to the holograms of the SSOV in advance based on Eq. (11) and the propagation distance *z*. Then when the beam irradiates the spinning object, the influence of the Gouy phase and diffraction is compensated to some extent, as shown in Figure 10. This method is helpful to improve the OAM mode purity of vortex beams in telemetry.

In short, we report a novel method to determine the azimuth of the rotating axis in real-time, which greatly improves the measurement efficiency. A proof-of-concept experiment is designed to prove the effectiveness and feasibility of the method. This method has great application potential in engineering practice. In addition, the idea of breaking circular symmetry and adding additional parameters of this scheme may be applied to particle manipulation, optical communication, remote sensing, and other fields.
